# Preparation and Characterization of Gelatin and Antioxidant Peptides from Gelatin Hydrolysate of Skipjack Tuna (*Katsuwonus pelamis*) Bone Stimulated by in vitro Gastrointestinal Digestion

**DOI:** 10.3390/md17020078

**Published:** 2019-01-24

**Authors:** Xiu-Rong Yang, Yu-Qin Zhao, Yi-Ting Qiu, Chang-Feng Chi, Bin Wang

**Affiliations:** 1 Zhejiang Provincial Engineering Technology Research Center of Marine Biomedical Products, School of Food and Pharmacy, Zhejiang Ocean University, 1st Haidanan Road, Zhoushan 316022, China; yxr1948008999@163.com (X.-R.Y.); zhaoy@hotmail.com (Y.-Q.Z.); qytdezh@icloud.com (Y.-T.Q.); 2 National and Provincial Joint Laboratory of Exploration and Utilization of Marine Aquatic Genetic Resources, National Engineering Research Center of Marine Facilities Aquaculture, School of Marine Science and Technology, Zhejiang Ocean University, 1st Haidanan Road, Zhoushan 316022, China

**Keywords:** Skipjack tuna (*Katsuwonus pelamis*), bone, gelatin, peptide, antioxidant activity

## Abstract

In China, a large amount of fish bones are produced during the processing of tuna cans production. For full use of those by-products, gelatin (STB-G) with a yield of 6.37 ± 0.64% was extracted from skipjack tuna (*Katsuwonus pelamis*) bone using water at 60 °C for 8 h. Amino acid analysis showed that STB-G contained Gly (340.3 residues/1000 residues) as the major amino acid and its imino acid content was 177.3 residues/1000 residues. Amino acid composition, SDS-PAGE, and Fourier transform infrared (FTIR) spectrum investigations confirmed that the physicochemical properties of STB-G were similar to those of type I collagen from skipjack tuna bone (STB-C), but partial high molecular weight components of STB-G were degraded during the extraction process, which induced that the gelatin was easier to be hydrolyzed by protease than mammalian gelatins and was suitable for preparation of hydrolysate. Therefore, STB-G was hydrolyzed under in vitro gastrointestinal digestion (pepsin-trypsin system) and five antioxidant peptides were purified from the resulted hydrolysate (STB-GH) and identified as GPDGR, GADIVA, GAPGPQMV, AGPK, and GAEGFIF, respectively. Among the gelatin hydrolysate, fractions, and isolated peptides, GADIVA and GAEGFIF exhibited the strongest scavenging activities on 2,2-diphenyl-1-picrylhydrazyl (DPPH) radical (EC_50_ 0.57 and 0.30 mg/mL), hydroxyl radical (EC_50_ 0.25 and 0.32 mg/mL), superoxide anion radical (EC_50_ 0.52 and 0.48 mg/mL), and 2,2′-azino-bis-3-ethylbenzothiazoline-6-sulfonic acid (ABTS) radical (EC_50_ 0.41 and 0.21 mg/mL). Moreover, GADIVA and GAEGFIF showed a high inhibiting ability on lipid peroxidation in a linoleic acid model system. The strong activities of five isolated peptides profited by their small molecular sizes and the antioxidant amino acid residues in their sequences. These results suggested that five isolated peptides (STP1–STP5), especially GADIVA and GAEGFIF, might serve as potential antioxidants applied in health food industries.

## 1. Introduction

Gelatin is produced by partial hydrolysis of collagen with a molecular weight (MW) ranging between 80 and 250 kDa [1]. Traditionally, gelatin has been isolated from the skin and bone collagens of mammalian species, primarily cows and pigs [2]. However, the application of gelatin from mammalian species may be a concern among consumers because of dietary restrictions and infectious disease [3]. These sociocultural and safety concerns have led to intensive research to identify and develop alternatives to mammal derived gelatin [1]. Gelatin from seafood by-products has been thought as an ideal alternative to mammalian gelatin due to its large quantities and safety [4]. In addition, gelatin from marine resources is acceptable for Islam, and can be used with minimal restrictions in Judaism and Hinduism [5]. Therefore, gelatin has been extensively extracted from different fish by-products, such as golden carp skin [2], lizardfish scale [6], unicorn leatherjacket skin [7], cuttlefish skin [8], cod head [9], shark cartilage [10], and red snapper and grouper bones [11].

Nowadays, fish gelatin is wildly applied in the food, pharmaceuticals, cosmetics, biomedical, and biomaterial-based packaging industries for its high nutrition, good moisture-retention, and biocompatibility [2,12]. However, its properties, including the lower melting temperature and gel strength, and weaker structural stability compared to mammal gelatin, severely limit its application extensively in tissue engineering materials and capsule wall materials [13,14]. Nevertheless, gelatin with those undesirable physicochemical properties is more easily hydrolyzed by proteases compared to mammal gelatin and is suitable to be prepared for bioactive peptides. Therefore, preparation of bioactive peptides using gelatins attracts broad attention due to its utilization potentiality in biomedical industries. Wu et al. reported that peptides of GPAGPHGPPGKDGR, AGPHGPPGKDGR, and AGPAGPAGAR from Pacific cod skin gelatin exhibited a high affinity to ferrous ions, which has a potential application of gelatin-derived peptides as novel carriers to combat iron deficiency [12]. Sun et al. evaluated the effect of tilapia gelatin peptides (TGP) on UV-induced damages to mice skin, and the results indicated that TGP could significantly prevent a decrease of antioxidase activity in a dose-dependent manner. The protective effects of TGP suggested that TGP could be a novel antiphotoaging agent from natural resources [15]. Chen and Hou reported that oral administration of gelatin hydrolysate from Pacific cod skin could suppress ultraviolet (UV) radiation-induced damage to the skin by inhibiting the depletion of endogenous antioxidant enzyme activity, and suppressing the expression of nuclear factor-κB (NF-κB) and NF-κB-mediated expression of pro-inflammatory cytokines [16]. Zheng et al. reported that gelatin hydrolysate (FSGH) of Nile tilapia skin prepared using ginger protease exhibited a higher degree of hydrolysis (DH), radical scavenging activity, and lipid peroxidation inhibition capability than those of gelatin hydrolysate from pig skin, pig bone, and bovine skin. Furthermore, tripeptides of Gly-Pro-Ala from FSGH can activate the expression of antioxidant response element (ARE)-driven antioxidant enzyme genes in a dose dependent manner and suppressed the H_2_O_2_-induced intracellular reactive oxygen species (ROS) production in the porcine small intestinal epithelial cell line (IPEC-J2) [17]. Therefore, gelatin peptides derived from seafood by-products exhibit significant antioxidant activity to protect the skin from photoaging through alleviating UV-induced abnormal changes of antioxidant defense systems, repairing endogenous collagen and elastin protein fibers, and decreasing the loss of moisture and lipids.

Skipjack tuna (*Katsuwonus pelamis*) belongs to the family of Scombridae, which is an epipelagic oceanic species widely distributed in subtropical and tropical seas [18]. It is the principal species of tropical tuna, with about 2.16 million metric tons caught in the Pacific Ocean in 2015 [18]. In China, a large amount of fish bones (spines and skulls) and skins are produced during the processing of tuna cans production [3], which contain about 30% collagen. Therefore, the preparation of collagen, gelatin, and bioactive peptides from tuna bones and skins could be a promising means to gain a value-added product and to lower environmental pollution. Shyni et al. extracted the skin gelatin of skipjack tuna and measured its physical and chemical properties, which indicated that its gelling temperature is low [19]. Yu et al. prepared acid- and pepsin-soluble collagens from the spine and skull of the skipjack tuna, and the data of the amino acid composition, SDS-PAGE pattern, and FTIR spectrum confirmed that acid-soluble collagen were type I collagen [3]. However, there was little information available about the preparation of gelatin and bioactive peptides using skipjack tuna bones. Therefore, the aims of this work were to (i) prepare and characterize gelatin from skipjack tuna bones (STB-G), (ii) isolate and identify the antioxidant peptides from hydrolysate of STB-G, and (iii) evaluate the in vitro antioxidant activities of isolated peptides form hydrolysate of STB-G.

## 2. Results and Discussion

### 2.1. Characterization of Gelatin from the Bones of Skipjack Tuna (STB-G)

#### 2.1.1. Yield and Proximate Composition of STB-G

The yield of STB-G was 6.37 ± 0.64% (on a wet bone weight basis), which was significantly lower than that (11.3 ± 0.03%) of gelatin from skipjack tuna skins [19]. The protein, moisture, ash, and fat contents of STB-G were 90.14 ± 3.98 g/100 g, 7.68 ± 0.41 g/100 g, 0.88 ± 0.15 g/100 g, and 0.68 ± 0.07 g/100 g, respectively. The protein content was higher than that (88.4 ± 0.12 g/100 g) of gelatin from skins of skipjack tuna [19]. The results indicated that the impurities in the bones of skipjack tuna were effectively removed through the extraction process of gelatin.

#### 2.1.2. Amino Acid Composition of STB-G

The amino acid composition and MW distribution are two key factors that influence the properties of gelatin [20]. As shown in Table 1, gelatin (STB-G) and Type I collagen (STB-C) from the bones of skipjack tuna had similar amino acid compositions. Glycine was the most abundant amino acid of STB-G and STB-C with contents of 340.3 and 339.1 residues/1000 residues, respectively, which is because approximately 50–60% of α-chains consist of typical tripeptide repetitions (Gly-X-Y), where X is mostly proline and Y is mainly hydroxyproline [21,22]. In addition, STB-G and STB-C were rich in alanine, proline, and hydroxyproline, in decreasing order, which is consistent with previous reports that those are the main amino acids in gelatins [20,23,24].

Hydroxyproline can form hydrogen bonds through its hydroxyl group to stabilize the triple-stranded collagen helix [22,25]. Pyrrolidine rings of imino acids (proline and hydroxyproline) are also confirmed to restrict the changes of the secondary structure of the polypeptide chain, and hence assist in reinforcing the thermal stability of the triple helical structure [14,26]. Therefore, the amount of imino acids highly affects the stability of the triple helix structure of the renatured gelatins [5,22]. As shown in Table 1, the content of imino acids of STB-G was 177.3 residues/1000 residues, which is similar to those of gelatins from the skin of dover sole (173–183 residues/1000 residues) [27] and bigeye snapper (186–187 residues/1000 residues) [28], but significantly lower than that of bovine gelatin (219.0 residues/1000 residues) [8]. Therefore, the helices of STB-G might be more unstable than those of bovine gelatin due to their lower contents of imino acids.

#### 2.1.3. Electrophoretic Pattern of STB-G

**Figure 1:**
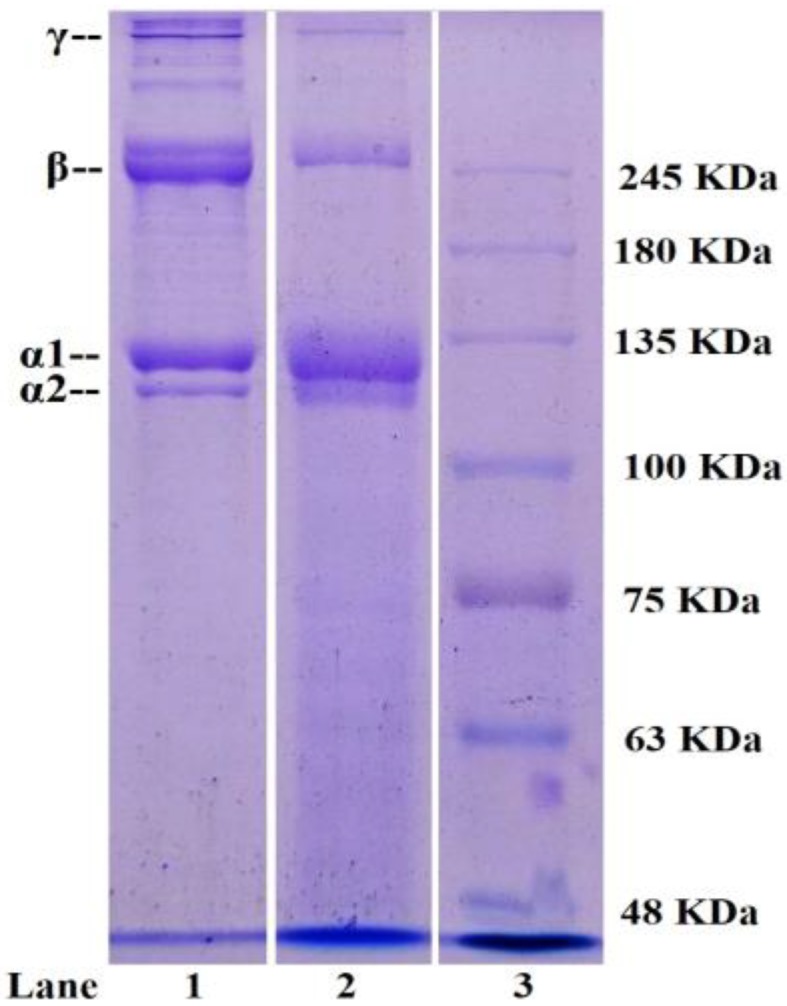
SDS-PAGE patterns of STB-G and STB-C analyzed by 7.5% separating gel and 4% stacking gel. Lane 1. Type I collagen from bones of skipjack tuna (STB-C); Lane 2. Gelatin from the bones of skipjack tuna (STB-G); Lane 3. Protein marker.

In addition to the amino acid composition, the distribution of the MW and composition of subunits also influences the properties of gelatins and collagens. SDS-PAGE patterns of STB-G and STB-C are shown in Figure 1. Similar protein patterns were observed from STB-G and STB-C, which consisted of two α-chains (α1 and α2 chains), and the band intensities of the α1-chain were approximately 2-fold higher than those of the α2-chain. The pattern was similar to type I collagen from seafood by-products [21,25]. It is generally known that type I collagen consists of two α1- and one α2-chain as the major component ([α1]_2_α2).

High MW components, particularly β (dimmers) and γ (trimers) components, were also observed in the protein patterns of STB-G and STB-C, but the β and γ band intensities of STB-C were significantly stronger than those of STB-G. In addition, the presence of peptides with MW below 100 kDa were slightly noticeable in STB-G. Those results indicated that heat induced cleavages of protein chains of STB-G during the extraction process [19,29].

#### 2.1.4. Fourier transform infrared (FTIR) Spectrum of STB-G

**Figure 2:**
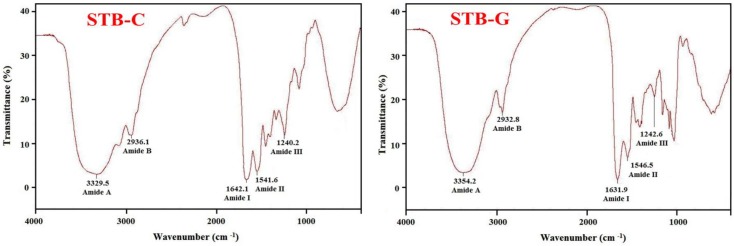
Fourier transform infrared (FTIR) spectra of STB-G and STB-C from the bones of skipjack tuna (*K. pelamis*).

As shown in Figure 2, STB-G and STB-C exhibited similar FTIR spectral profiles. The spectrum of STB-G had major peaks associated with amide bands I (1631.9 cm^−1^), II (1546.5 cm^−1^), III (1242.6 cm^−1^), A (3354.2 cm^−1^), and B (2932.8 cm^−1^), which were similar to those of gelatins from the skins of seabass [30], golden carp [2], and brownbanded bamboo shark and blacktip shark [31].

Amide I, II, and III bands are closely related to the triple helical structure of gelatin and collagen, resulting from C=O stretching, N–H bending, and C–H stretching, respectively [5]. Amide I bands occurring in the range of 1600–1700 cm^−1^ are primarily associated with C=O stretching vibration along the polypeptide backbone or a hydrogen bond coupled with COO–, and a reduction of the molecular order will result in a peak shift to a lower wavenumber [2,14]. Yakimets et al. (2005) reported that the absorption wavenumber of the amide I band at 1634.7 cm^−1^ represented gelatin with a distinctive coiled conformation [32]. The lower wavenumber of STB-G (1631.9 cm^−1^), compared to that of STB-C (1642.1 cm^−1^), indicated that C=O stretching vibration was reduced and partial telopeptides were degraded by heating during the preparation process [21]. The amide II band with wavenumbers ranging from 1500 to 1600 cm^−1^ specifies the number of N–H groups involved in hydrogen bonding with the adjacent α-chain; therefore, a lower wavenumber of the amide II band indicates both increased hydrogen bonding by N–H groups, and a higher structure order [5,14]. The wavenumbers of STB-G and STB-C were found to be 1546.5 and 1541.6 cm^−1^, respectively. The present data indicated that there was less hydrogen bonding in STB-G than that in STB-C. Amide III absorption, which is associated with the triple helix structure of collagen and is involved in C–N stretching and N–H in plane bending from amide linkages, is normally very weak in FTIR. It also arises from wagging vibrations of CH_2_ groups from the glycine backbone and proline side-chains [26]. In this study, the amide III bands of STB-G and STB-C were located at wavenumbers of 1242.6 cm^−1^ and 1240.2 cm^−1^, respectively. The higher amplitude was suggested to be due to the conversion of the α-helix structure to random coils upon heating. The changes were linked to the denaturation of collagen to gelatin with a loss in the triple helical structure [2].

Normally, stretching vibration of free N–H groups occurs within the range of 3400–3440 cm^−1^ [21]. The wavenumber shifts to a lower frequency when the N–H group of the peptide is involved in hydrogen bonding [14]. Figure 2 shows that the amide A wavenumbers of STB-C (3329.5 cm^−1^) and STB-G (3354.2 cm^−1^) indicate that some N–H groups in STB-G and STB-C contributed to the formation of hydrogen bonds, and the degree of hydrogen bonding in STB-C was more than that of STB-G. The amide B band is related to asymmetric stretch vibrations of –${\mathrm{NH}}_{3}^{+}$ and =C–H, and the shift of amide B to a higher wavenumber is associated with an increase in free NH–${\mathrm{NH}}_{3}^{+}$ groups from both lysine residues and the *N*-terminus [5,14]. The wavenumbers of the amide B band of STB-C and STB-G were found at 2936.1 cm^−1^ and 2932.8 cm^−1^, respectively, indicating STB-C had fewer free –${\mathrm{NH}}_{3}^{+}$ groups than STB-G.

### 2.2. Purification of Antioxidant Peptides from Hydrolysate of STB-G

#### 2.2.1. Preparation and Fractionation of Hydrolysate from STB-G

The ideal in vitro digestion method can provide a useful alternative to animal and human models and serve as a tool for rapid screening foods or delivery systems with different compositions and structures [33]. Therefore, in vitro gastrointestinal (GI) digestion models are usually applied to prepare protein hydrolysates due to their rapid, inexpensive, and safe properties [34,35]. STB-G was hydrolyzed under in vitro GI digestion (pepsin-trypsin system), and the resulting hydrolysate (referred to as STB-GH) with a degree of hydrolysis of 23.78 ± 1.24% could strongly scavenge DPPH radical with a half elimination ratio (EC_50_) value of 3.28 mg protein/mL, which is lower than those of gelatin (collagen) hydrolysates from tilapia skin (EC_50_ value of 3.66 mg/mL) [36], skate (*Raja porosa*) cartilage (EC_50_ value of 13.13 mg protein/mL) [37], and bluefin leatherjacket skin (EC_50_ value of 5.23 mg protein/mL) [38] and heads (15.98% at 10 mg/mL) [39], but higher than those of gelatin (collagen) hydrolysates from salmon pectoral fin (EC_50_ value of 1.63 mg /mL) [34] and thornback ray (EC_50_ value of 1.98 mg/mL) [40].

Gelatin hydrolysates contain multiple peptides with different chain lengths and amino acid compositions. Hydrolysate fractions with smaller MW showed stronger antioxidant activity than those of larger MW hydrolysates because peptides with a short chain length are more accessible to free radicals and allow them to more easily trap the free radical [41,42]. For enrichment of functional peptides, STB-GH was further divided into three fractions, including STB-GH-I (<3 kDa), STB-GH-II (3–5 kDa), and STB-GH-III (>5 kDa), by ultrafiltration with MW Cut Off (MWCO) membranes of 3 and 5 kDa. As shown in Table 2, the EC_50_ value of STB-GH-I on the DPPH radical was 1.84 mg protein/mL, which was significantly stronger than those of STB-GH (3.28 mg protein/mL), STB-GH-II (4.36 mg protein/mL), and STB-GH-III (>10 mg protein/mL) (*p* < 0.05). This data was in line with previous reports that the antioxidant abilities of protein hydrolysates were negatively correlated with their average MW [43]. Therefore, STB-GH-I was selected for the subsequent chromatographic separation.

#### 2.2.2. Anion-Exchange Chromatography

**Figure 3:**
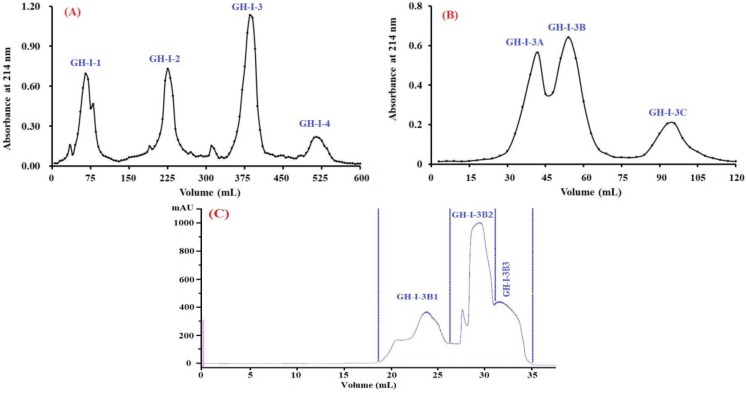
Elution profile of STB-GH-I in diethylaminoethanol (DEAE)-52 cellulose anion-exchange chromatography (**A**), GH-I-3 in Sephadex G-25 chromatography (**B**), and GH-I-3B in a Superdex^®^ Peptide 10/300 GL column (**C**).

Acidic and hydrophobic amino acid residues in peptide sequences can be absorbed by the anion-exchange resins on hydrogen bonds and/or van der Waals forces [44]. Therefore, anion exchange resins are usually applied to isolated bioactive peptides and proteins from solutions. As shown in Figure 3A, four fractions (GH-I-1 to GH-I-4) were separated from STB-GH-I using a DEAE-52 cellulose column. Amongst them, GH-I-1 was eluted using deionized water (DW), and GH-I-2, GH-I-3, and GH-I-4 were eluted using 0.1, 0.5, and 1.0 M NaCl, respectively. EC_50_ values of STB-GH-I and its four fractions on the DPPH radical are shown in Table 2, and the data demonstrated that GH-I-3 with an EC_50_ value of 1.32 mg protein/mL showed significantly stronger DPPH radical scavenging activity than those of STB-GH-I (EC_50_ value of 1.84 mg protein/mL), GH-I-1 (EC_50_ value of 8.73 mg protein/mL), GH-I-2 (EC_50_ value of 3.47 mg protein/mL), and GH-I-4 (EC_50_ value of 3.41 mg protein/mL) (*p* < 0.05). Thus, GH-I-3 was selected for the following experiment.

#### 2.2.3. Gel Filtration Chromatography (GFC)

GFC is a popular method for the preparation of proteins or peptides on their molecular size used in food products and pharmaceutical industries [41]. As shown in Figure 3B, GH-I-3 was further divided into three fractions (GH-I-3A to GH-I-3C) using a Sephadex G-25 column. The EC_50_ value of GH-I-3B on the DPPH radical was 1.08 mg protein/mL, which was significantly higher than those of GH-I-3 (EC_50_ value of 1.32 mg protein/mL), GH-I-3A (EC_50_ value of 3.69 mg protein/mL), and GH-I-3C (EC_50_ value of >10 mg protein/mL) (*p* < 0.05). Therefore, fraction GH-I-3B was further separated into three components (GH-I-3B1 to GH-I-3B3) using a Superdex^®^ Peptide 10/300 GL column (300 mm × 10 mm, 13–15 μm) in the experiment (Figure 3C). As shown in Table 2, the EC_50_ value of GH-I-3B2 on the DPPH radical was 0.87 mg protein/mL, which was significantly higher than those of GH-I-3B (EC_50_ value of 1.08 mg protein/mL), GH-I-3B1 (EC_50_ value of 2.68 mg protein/mL), and GH-I-3B3 (EC_50_ value of 5.74 mg protein/mL) (*p* < 0.05). Therefore, GH-I-3B2 should contain strong antioxidant peptides and was suitable for the following separation process.

#### 2.2.4. Purification of Peptides from GH-I-3B by Reverse-Phase High Performance Liquid Chromatography (RP-HPLC)

**Figure 4:**
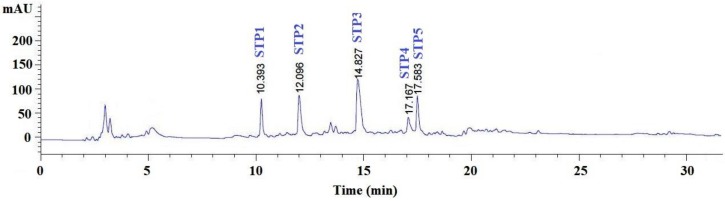
Elution profile of GH-I-3B separated by the reversed-phase high-performance liquid chromatography (RP-HPLC) on a Zorbax, SB C-18 column (4.6 mm × 250 mm) from 0 to 35 min.

RP-HPLC is an effective technique applied to purify bioactive peptides in a hydrolysate mixture on their hydrophobic character [39]. As shown in Figure 4, GH-I-3B was finally purified using the RP-HPLC system on an Agilent 1260 HPLC system with a Zorbax C-18 column, and the eluted peptides were gathered separately on the chromatographic peaks. At last, five peptides with a retention time of 10.393 min (STP1), 12.096 min (STP2), 14.827 min (STP3), 17.168 min (STP4), and 17.583 min (STP5) were collected and lyophilized for amino acid sequence identification and activity evaluation.

### 2.3. Amino Acid Sequence Analysis and Mass Spectrometry of Peptides from Gelatin Hydrolysate of Skipjack Tuna (K. pelamis) Bone

The amino acid composition, sequences, and molecular mass of five isolated peptides (STP1–STP8) were determined using a protein sequencer and electrospray ionization mass spectrometry (ESI-MS), and the results are shown in Table 3. The amino acid sequences of five gelatin peptides (STP1–STP5) were identified as Gly-Pro-Asp-Gly-Arg (GPDGR, STP1), Gly-Ala-Asp-Ile-Val-Arg (GADIVA, STP2), Gly-Ala-Pro-Gly-Pro-Glu-Met-Val (GAPGPQMV, STP3), Ala-Gly-Pro-Lys (AGPK, STP4), and Gly-Ala-Glu-Gly-Phe-Ile-Phe (GAEGFIF, STP5) with MWs of 500.43, 544.55, 756.84, 374.39, and 739.76 Da, respectively, which agreed well with their theoretical masses (Table 3).

### 2.4. Antioxidant Activity

**Figure 5:**
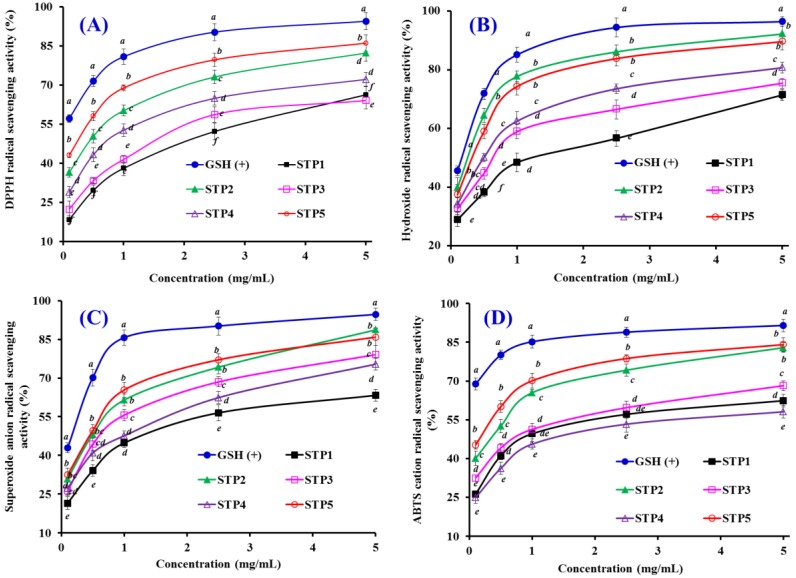
2,2-diphenyl-1-picrylhydrazyl (DPPH) radical (**A**), hydroxyl radical (**B**), superoxide anion radical (**C**), and 2,2′-azinobis-(3-ethylbenzothiazoline-6-sulfonic acid) (ABTS) cation radical (**D**) scavenging activities of five antioxidant peptides (STP1-STP5) from gelatin hydrolysate of skipjack tuna (*K. pelamis*) bone. All data are presented as the mean ± SD of triplicate results. ^a–f^ Values with the same letters indicate no significant difference of different samples at the same concentrations (*p* > 0.05).

**Figure 6:**
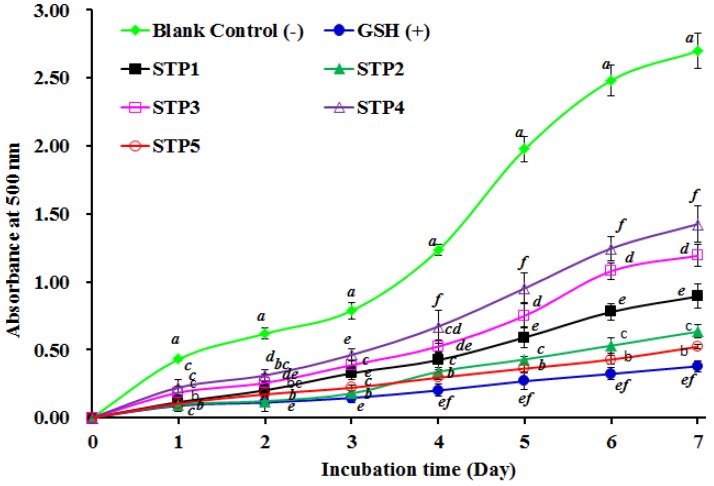
Lipid peroxidation inhibition assays of five antioxidant peptides (STP1–STP5) from gelatin hydrolysate of skipjack tuna (*K. pelamis*) bone. All data are presented as the mean ± SD of triplicate results. ^a–f^ Values with the same letters indicate no significant difference of the different samples at the same concentrations (*p* > 0.05).

To better evaluate the antioxidant activity of five isolated peptides (STP1–STP5) from gelatin hydrolysate of skipjack tuna bone, four kinds of radical scavenging assays and lipid peroxidation inhibition assay were tested, and the results are presented in Table 4 and Figure 5 and Figure 6.

#### 2.4.1. Radical Scavenging Activity

##### DPPH Radical Scavenging Activity

DPPH is a stable and cell-permeable radical that is commonly applied to measure the antioxidant ability of peptides, which serve as hydrogen donor or free radical scavenger. The reaction of DPPH with an antioxidant peptide produces the corresponding hydrazine, DPPH2, with the color of the reaction solution changing from purple (absorbance at around 520 nm) to yellow [44]. As shown in Figure 5A, five antioxidant peptides (STP1–STP5) showed strong DPPH radical scavenging activities with a positive correlation between the concentration and the activity, but their activity was still lower than that of the positive control of GSH at the same concentration. The EC_50_ value of STP5 was 0.30 mg/mL, which was significantly lower than those of STP1 (2.49 mg/mL), STP2 (0.57 mg/mL), STP3 (1.93 mg/mL), and STP4 (1.66 mg/mL), respectively. In addition, The EC_50_ values of STP2 and STP5 were lower than those of most antioxidant peptides from protein hydrolysates of salmon pectoral fin (TTANIEDRR: 2.503 mg/mL) [45], skate cartilages (FIMGPY: 2.60 mg/mL; GPAGDY: 3.48 mg/mL; IVAGPQ: 3.93 mg/mL) [37], scalloped hammerhead cartilage (GPE: 2.43 mg/mL; GARGPQ: 2.66 mg/mL; GFTGPPGFNG: 1.99 mg/mL) [46], swim bladders of miiuy croaker (GIEWA: 0.78 mg/mL) [47], and blue mussel (YPPAK: 2.62 mg/mL) [48]. However, the EC_50_ values of STP2 and STP5 were higher than those of peptides from protein hydrolysates of grass carp skin (HFGBPFH: 0.20 mg/mL) [49] and skate muscle (NWDMEKIWD 0.289 mg/mL) [50]. Therefore, five gelatin peptides (STP1 to STP5), especially STP2 and STP5, had a strong ability to serve as a hydrogen donor or free radical scavenger for preventing the DPPH radical reaction.

##### Hydroxyl Radical Scavenging Activity

Hydroxyl radicals are able to instantaneously attack and unselectively oxidize biomacromolecules, initiating the process of oxidative stress in an organism. Therefore, it is one of the important ways to search for antioxidant agents. As presented in Figure 5B, five peptides (STP1–STP5) showed dose-related effects in the scavenging activity of hydroxyl radicals at peptide concentrations ranging from 0 to 5.0 mg/mL. The EC_50_ values of STP1, STP2, STP3, STP4, and STP5 were 1.21, 0.25, 0.64, 0.49, and 0.32 mg/mL, respectively, and STP2 exhibited the highest hydroxyl radical scavenging ability among all isolated peptides at the same concentration, but its activity was still lower than that of the positive control of GSH. The EC_50_ values of STP2 and STP5 were lower than those of most peptides from protein hydrolysates of conger eel (LGLNGDDVN: 0.687 mg/mL) [51], giant squid (NADFGLNGLEGLA: 0.612 mg/mL) [52], swim bladders of miiuy croaker (FPYLRH: 0.68 mg/mL; GIEWA: 0.71 mg/mL) [47], grass carp skin (PYSFK: 2.283mg/mL; VGGRP: 2.055 mg/mL) [49], and weatherfish loach (PSYV: 2.64 mg/mL) [53]. Nevertheless, EC_50_ values of STP2 and STP5 were still higher than those of antioxidant peptides from spotless smoothhound cartilage (AEVG: 0.06 mg/mL) [54], blue mussel (YPPAK: 0.228 mg/mL) [48], and skate muscle (NWDMEKIWD 0.176 mg/mL) [50]. The data indicated that STP2 and STP5 could act as a scavenger to decrease the hydroxyl radical damage in biological systems.

##### Superoxide Anion Radical Scavenging Assay

Superoxide anion radical can undergo fenton-chemistry and produce the highly reactive hydroxyl radical to inactivate enzymes with an iron-sulfur cluster, initiate lipid peroxidation, and react with carbonyl compounds to generate toxic peroxy radicals. Superoxide anion radicals are catalyzed into hydrogen peroxide and oxygen by superoxide dismutases (SOD) in an organism. Figure 5C indicates that five antioxidant peptides (STP1–STP5) showed strong superoxide anion radical scavenging activities in a dose-effect manner with EC_50_ values of 1.48, 0.52, 0.67, 1.22, and 0.48 mg/mL, respectively, but their activities were still lower than that of GSH at a concentration ranging from 0.1 to 5.0 mg/mL. The EC_50_ values of STP2, STP3, and STP5 were lower than those of peptides from protein hydrolysates of miiuy croaker swim bladders (GFEPY: 0.87 mg/mL; FYKWP: 1.92 mg/mL; FTGMD: 3.04 mg/mL; GFYAA: 3.03 mg/mL; FSGLR: 3.35 mg/mL) [47], croceine croaker muscle (VLYEE: 0.693 mg/mL; MILMR: 0.993 mg/mL) [55], bluefin leatherjacket heads (WEGPK: 3.223 mg/mL; GPP: 4.668 mg/mL; GVPLT: 2.8819 mg/mL) [39], and skate cartilage (FIMGPY: 1.61 mg/mL; GPAGDY: 1.66 mg/mL; IVAGPQ: 1.82 mg/mL) [37]. However, the EC_50_ values of STP2, STP3, and STP5 were higher than those of peptides from protein hydrolysates of croceine croaker muscle (YLMR: 0.450 mg/mL) [55], swim bladder of miiuy croakers (FPYLRH: 0.34 mg/mL; GIEWA: 0.30 mg/mL) [47], round scad (HDHPVC: 0.265 mg/mL; HEKVC: 0.235 mg/mL) [56], monkfish muscle (FLHRP: 0.101 mg/mL; LMGQW: 0.042 mg/mL) [57], and croceine croaker scales (GFRGTIGLVG: 0.463 mg/mL; GPAGPAG: 0.099 mg/mL; GFPSG: 0.151 mg/mL) [58]. Therefore, STP2, STP3, and STP5 can serve as superoxide anion radical scavengers to eliminate radical damage together with SOD in organisms.

##### 2,2′-azino-bis-3-ethylbenzothiazoline-6-sulfonic acid (ABTS) Cation Radical Scavenging Assay

2,2′-azino-bis-3-ethylbenzothiazoline-6-sulfonic acid (ABTS) is a popular measure of the antioxidant capacities of peptides because ABTS is converted to its radical cation by the addition of sodium persulfate, which shows a blue color with the absorbance at 734 nm. As shown in Figure 5D, the ABTS cation radical scavenging ratios of five antioxidant peptides (STP1–STP5) increased with an increasing concentration ranging from 0.1 to 5.0 mg/mL, but their activities were still lower than that of GSH at the same concentration. The EC_50_ values of STP2 and STP5 were 0.41 and 0.21 mg/mL, respectively, which were significantly lower than those of the other three peptides. Furthermore, the EC_50_ values of STP2 and STP5 were lower than those of peptides from protein hydrolysates of salmon (FLNEFLHV: 1.548 mg/mL) [44], skate cartilages (FIMGPY: 1.04 mg/mL; GPAGDY: 0.77 mg/mL; IVAGPQ: 1.29 mg/mL) [37], bluefin leatherjacket heads (WEGPK: 5.407 mg/mL; GPP: 2.472 mg/mL; GVPLT: 3.124 mg/mL) [39], and grass carp skin (VGGRP: 0.465 mg/mL) [49]). These results indicated that the five antioxidant peptides (STP1–STP5), especially STP2 and STP5, could effectively inhibit the ABTS cation radical chain reaction by converting it to the colorless form.

#### 2.4.2. Lipid Peroxidation Inhibition Assay

The oxidative process is complicated in food and biological systems and embroiled in multifarious reactions for the propagation of lipid radicals hydroperoxides [41,47]. As a consequence, we used the lipid peroxidation inhibition assay in a linoleic acid model system to determine the antioxidant abilities of five antioxidant peptides (STP1–STP5). As presented in Figure 6, the absorbance values at 500 nm of the STP2 and STP5 solutions were significantly lower than that of the blank control (without antioxidant) and the other three peptides (STP1, STP3, and STP4). Furthermore, the absorbance values at 500 nm of the STP2 and STP5 solutions were a little higher than that of the positive control of GSH. The data indicated that the abilities of STP2 and STP5 on lipid oxidation inhibition were similar to that of GSH in the tested system during 7 days incubation.

#### 2.4.3. Relationship among the Molecular Size, Amino Acid Composition, and Antioxidant Activity of STP1–STP5

Molecular size and amino acid composition and sequence are thought of as the key roles in the antioxidant capacities of peptides [41]. In the study, the five antioxidant peptides (STP1–STP5) from the gelatin hydrolysate of skipjack tuna bone are tetrapeptide to octapeptide with MWs ranging from 374.39 Da to 756.84 Da (Table 3). The data indicated that the five isolated peptides have a higher possibility of interacting with free radicals to prevent lipid peroxidation [42,43].

Hydrophobic amino acids, such as Pro, Leu, Met, Ala, Val, and Ile, have a high reactivity to hydrophobic PUFAs and exert their significant effects on radical scavenging in lipid-rich foods [41,59]. Therefore, the hydrophobic amino acid resides of Ala, Ile, and Val in STP2, and Ala and Ile in STP5 will help antioxidant peptides more easily contact with target free radicals. Moreover, aromatic groups of Phe, Trp, and Tyr can keep radicals stable during the scavenging process by contributing protons to electron deficient radicals [60]. Therefore, Phe residues in the sequences of STP5 should contribute to its antioxidant activity.

Polar amino acids are reported to play a critical role in hydroxyl radical scavenging and metal ion chelating activities because of their carboxyl and amino groups in the side chains [61,62]. Zhu et al. reported that peptides consisting of Glu, Lys, and Asp have strong abilities to chelate metal ions as well as scavenge hydroxyl radicals [62]. Ren et al. reported that Arg had a greater capacity to scavenge hydroxyl radicals [63]. A single hydrogen atom of Gly residue can provide high flexibility to the peptide backbone and serve as a proton-donator to neutralize active free radicals [46,64]. Therefore, polar amino acids, including Gly, Asp, and Arg residues in STP2, and Gly and Glu residues in STP5, could play a critical role in their radical scavenging and lipid peroxidation inhibition activities.

## 3. Experimental Section

### 3.1. Materials

Bones of kipjack tuna (*K. pelamis*) were kindly supplied by Zhejiang Hailisheng Group Co. Ltd. (Zhejiang, China). Type I collagen from the bones of skipjack tuna (STB-C) was prepared by our lab. DEAE-52 cellulose, bovine serum albumin (BSA), and Sephadex G-25 were purchased from Shanghai Source Poly Biological Technology Co., Ltd (Shanghai, China). Acetonitrile (ACN) of liquid chromatogram grade and trifluoroacetic acid (TFA) were purchased from Thermo Fisher Scientific Co., Ltd (Shanghai, China). DPPH, Superdex^®^ Peptide 10/300 GL column, and ABTS were purchased from Sigma–Aldrich (Shanghai) Trading Co., Ltd. (Shanghai, China). GPDGR (STP1), GADIVA (STP2), GAPGPEMV (STP3), AGPM (STP4), and GAEGFIF (STP5) with purity higher than 98% were synthesized in China Peptides Co. (Suzhou, China). All other reagents were analytical grade and purchased from Sinopharm Chemical Reagent Co., Ltd. (Shanghai, China).

### 3.2. Preparation of Gelatin (STB-G) and Gelatin Hydrolysate (STB-GH) of Kipjack Tuna Bone

Bones of kipjack tuna were prepared following a previously established protocol [3]. Briefly, frozen bones were unfrozen and processed into small pieces (1–2 cm). Then, bone debris were mixed with 0.1 M NaOH at a bone/solution ratio of 1:10 (*w*/*v*) for 6 h, and the solution was replaced every 2 h to remove non-collagenous proteins. The alkaline-bone pieces were washed with tap water at a ratio of 1:20 (*w*/*v*) for 3 times. After that, the bone pieces were demineralized with 0.5 M EDTA-2Na (pH 7.4) at a ratio of 1:10 (*w*/*v*) for 12 h, and the solution was changed every 12 h. Then, the demineralized-bone pieces were washed with tap water at a ratio of 1:20 (*w*/*v*) for 10 min and the washing was performed 3 times. The demineralized-bone pieces were then soaked in 0.2 M acetic acid solution with a bone/solution ratio of 1:10 (*w*/*v*) for 24 h, and acidic solution was changed every 12 h to swell the collagenous material in the bone. Acid treated bone pieces were washed with tap water until the wash water became neutral and finally washed with distilled water (DW) to remove other residues.

The gelatin was extracted from pretreated bone pieces of kipjack tuna using the method described by Shyni et al. with a slight modification [19]. The gelatin extraction was carried out in DW at 60 °C for 8 h with a bone/water ratio of 1:10 (*w*/*v*). Finally, the extracting solution was filtered with filter paper, and the filtrate was centrifuged at 12,000 g for 15 min. The resulting supernatant, named as STB-G, was obtained and lyophilized. The yield of gelatin was calculated on the dry matter of freeze-dried collagens in comparison with the wet weight of bones used for extraction.

STB-G hydrolyzed under in vitro gastrointestinal digestion (pepsin-trypsin system) was performed following the method of Phongthai et al. [35]. Gelatin dispersions (pH 1.5, 1%, *w*/*v*) were hydrolyzed using pepsin at pH 1.5, 37.0 °C with a total enzyme dose of 1% (*w*/*w*, 1 g enzyme/100 g gelatin). In 2 h, the mixture was neutralized with NaOH solution (1.0 M) and hydrolyzed using trypsin at pH 7.0, 37.0 °C with a total enzyme dose of 1% (*w*/*w*, 1 g enzyme/100 g gelatin) for 2 h. After that, the gelatin hydrolysate was heated at 90 °C for 15 min to terminate trypsin digestion and centrifuged at 8000 g for 15 min at room temperature. The resulting supernatant, referred to as STB-GH, was collected, freeze-dried, and kept at −20 °C for further analysis. The concentrations of STB-GH and its fractions were expressed as mg protein/mL and measured by the dye binding method of Bradford with BSA as the standard protein.

### 3.3. Characterization of Gelatin (STB-G)

#### 3.3.1. Proximate Analysis

Moisture, ash, fat, and protein contents of the skull, spine, and collagen were determined according to the methods of Association of Official Analytical Chemist (AOAC) with the method numbers of 950.46B, 920.153, 960.39 (a), and 928.08, respectively [65].

#### 3.3.2. Determination of Amino Acid Composition

Amino acid analysis was measured according to the methods described by Zhao et al. [47]. STB-G was dissolved in DW to obtain a final concentration of 1 mg/ml, and an aliquot of 50 mL was dried and hydrolyzed in vacuum-sealed glass tubes at 110 °C for 24 h in the presence of constant boiling of 6 mM HCl containing 0.1% phenol and using norleucine as the internal standard. After hydrolysis, samples were again vacuum-dried, dissolved in application buffer, and injected into an automated amino acid analyser (HITACHI 835-50 Amino Acid Analyzer, Tokyo, Japan).

#### 3.3.3. SDS-PAGE

Electrophoretic patterns of STB-G and STB-C were measured according to the previous method with a slight modification, using 7.5% separating gel and 4% stacking gel [14]. The samples (10 μg proteins) were mixed with the sample loading buffer (60 mM Tris-HCl, pH 8.0, containing 25% glycerol, 2% SDS, 0.1% bromophenol blue) at a 4:1 (*v*/*v*) ratio in the presence of β-ME, then applied to sample wells and electrophoresed in an electrophoresis instrument (AE-6200, ATTO Corporation, Japan). The electrophoresis was carried out for about 4 h at a constant voltage of 100 V. After electrophoresis, the gel was fixed with 50% (*v*/*v*) methanol and 10% acetic acid for 30 min. The gel was then stained for 3 h with 0.05% (*w*/*v*) Coomassie blue R-250 in 15% (*v*/*v*) methanol and 5% (*v*/*v*) acetic acid. The gel was finally destained with 30% (*v*/*v*) methanol and 10% (*v*/*v*) acetic acid. High MW marker was used to estimate the MWs of proteins. Type I collagen from the bones of skipjack tuna (STB-C) was used as a standard.

#### 3.3.4. FTIR

The infrared spectra (450–4000 cm^−1^) of STB-G and STB-C were recorded in KBr disks with a Fourier transform IR spectrophotometer (Nicolet 6700, Thermo Fisher Scientific Inc., Waltham, MA, USA). One milligram of dry sample was mixed with 100 mg of dry KBr, and the mixture was pressed into a disk for spectrum recording.

### 3.4. Isolation of Peptides from STB-GH

#### 3.4.1. Fractionation of STB-GH

STB-GH was fractionated using ultrafiltration (8400, Millipore, Hangzhou, China) with 3 and 5 kDa MWCO membranes (Millipore, Hangzhou, China), and three fractions termed STB-GH-I (MW <3 kDa), STB-GH-II (MW 3–5 kDa), and STB-GH-III (MW >5 kDa) were collected and lyophilized.

#### 3.4.2. Anion-Exchange Chromatography

STB-GH-I solution (5 mL, 40.0 mg/mL) was injected into a DEAE-52 cellulose column (1.6 cm × 80 cm) pre-equilibrated with DW, and stepwise eluted with 150 mL DW, 0.1 M NaCl, 0.5 M NaCl, and 1.0 M NaCl solution at a flow rate of 1.0 mL/min, respectively. Each eluate (5 mL) was monitored at 214 nm. Finally, five fractions (GH-I-1 to GH-I-4) were pooled and lyophilized on the chromatographic peaks.

#### 3.4.3. Gel Filtration Chromatography

GH-I-3 solution (5 mL, 20.0 mg/mL) was separated on a Sephadex G-15 column (2.6 cm × 160 cm) eluted with DW at a flow rate of 0.6 mL/min. Each eluate (3 mL) was collected and monitored at 214 nm, and the fraction of GH-I-3B solution (25 μL, 10.0 mg/mL) was further separated by ÄKTA avant 25 (GE Healthcare Life Sciences, Chicago, IL, USA) with a Superdex^®^ Peptide 10/300 GL column (300 mm × 10 mm, 13–15 μm) at a flow rate of 0.75 mL/min. The eluted DW was monitored at 214 nm, and three subfractions (GH-I-3A, GH-I-3B, and GH-I-3C) were collected and lyophilized.

#### 3.4.4. RP-HPLC

GH-I-3B2 was further purified on an Agilent 1260 HPLC system (Agilent Ltd., Santa Rosa, CA, USA) with a Zorbax, SB C-18 column (4.6 mm × 250 mm). The sample was eluated with a linear gradient of acetonitrile (0–50% in 0–35 min) in 0.1% TFA at a flow rate of 0.8 mL/min. Five antioxidant peptides (STP1 to STP5) were isolated on the absorbance at 214 nm and lyophilized.

### 3.5. Amino Acid Sequence and Molecular Mass Analysis

The amino acid sequences and molecular masses of five isolated peptides (STP1 to STP5) were measured on an Applied Biosystems 494 protein sequencer (Perkin Elmer/Applied Biosystems Inc., Foster City, CA, USA) and a Q-TOF mass spectrometer coupled with an electrospray ionization source, respectively.

### 3.6. Antioxidant Activity

The lipid peroxidation inhibition and radical scavenging assays of five isolated peptides (STP1 to STP5) were determined by the previous method [47], and the results of the radical scavenging assays were expressed as a half elimination ratio (EC_50_) defined as the concentration where a sample caused a 50% decrease of the initial concentration of DPPH radical, hydroxyl radical, superoxide anion radical, and ABTS cation radical, respectively. The calculation method of EC_50_ was according to the linear relationship of radical scavenging rates and concentrations of samples.

### 3.7. Statistical Analysis

The data are reported as the mean ± standard deviation (SD) with three determinations. A one-way analysis of variance (ANOVA) test for differences between the means of each group was applied to analyzed data using SPSS 19.0 (Statistical Program for Social Sciences, SPSS Corporation, Chicago, IL, USA). A *p*-value of less than 0.05 was considered statistically significant.

## 4. Conclusions

In the experiment, the gelatin (STB-G) of skipjack tuna (*K. pelamis*) bone was extracted using hot water and its physicochemical properties (SDS-PAGE, FT-IR, and amino acid composition) indicated that it was similar to collagen from skipjack tuna bone and was more suitable for preparation of hydrolysate than mammalian gelatin. Therefore, STB-G was hydrolyzed under in vitro gastrointestinal digestion and five antioxidant peptides were purified from the resultant hydrolysate and identified as GPDGR, GADIVA, GAPGPQMV, AGPK, and GAEGFIF, respectively. The five peptides exhibited high radical scavenging and lipid peroxidation inhibition capabilities, and their activities benefitted from their small molecular sizes and the antioxidant amino acid residues in their sequences. The present results indicated that gelatin hydrolysate and antioxidant peptides from skipjack tuna bones may be applied as an ingredient in new functional foods. In addition, cell and animal level experiments will be performed to discuss the antioxidant mechanism of the five antioxidant peptides.

## Figures and Tables

**Table 1:** Amino acid composition of gelatin (STB-G) and Type I collagen (STB-C) from the bones of skipjack tuna (residues/1000 residues).

Amino Acid	STB-G	STB-C
Hydroxyproline (Hyp)	72.5	73.8
Glutamic acid (Glu)	64.7	66.7
Aspartic acid (Asp)	46.1	46.8
Threonine (Thr)	25.6	25.2
Serine (Ser)	36.4	33.3
Proline (Pro)	104.8	104.4
Glycine (Gly)	340.3	339.1
Alanine (Ala)	125.3	126.3
Cysteine (Cys)	ND	ND
Valine (Val)	25.4	26.0
Methionine (Met)	14.7	14.5
Isoleucine (Ile)	11.4	12.7
Leucine (Leu)	25.2	26.0
Tyrosine (Tyr)	4.3	2.9
Phenylalanine (Phe)	13.8	14.3
Hydroxylysine (Hyl)	5.6	4.9
Lysine (Lys)	29.2	29.5
Histidine (His)	5.5	5.3
Arginine (Arg)	49.2	48.3
Total	1000.0	1000.0
Imino acid (Pro + Hyp)	177.3	178.2

ND = not detected.

**Table 2:** EC_50_ values of gelatin hydrolysate from the bones of skipjack tuna (STB-GH) and its fractions on 2,2-diphenyl-1-picrylhydrazyl (DPPH) radical.

Gelatin Hydrolysate and Fractions	EC_50_ Value (mg protein/mL)	Gelatin Hydrolysate and Fractions	EC_50_ Value (mg protein/mL)	Gelatin Hydrolysate and Fractions	EC_50_ Value (mg protein/mL)
STB-GH	3.28	GH-I-2	3.47	GH-I-3C	>10
STB-GH-I	1.84	GH-I-3	1.32	GH-I-3B1	2.68
STB-GH-II	4.36	GH-I-4	3.41	GH-I-3B2	0.87
STB-GH-III	>10	GH-I-3A	3.69	GH-I-3B3	5.74
GH-I-1	8.73	GH-I-3B	1.08		

All data are presented as the mean ± SD of triplicate results.

**Table 3:** Retention time, amino acid sequences, and molecular mass of five antioxidant peptides (STP1–STP5) from gelatin hydrolysate of skipjack tuna (*K. pelamis*) bone.

No.	Retention Time (min)	Amino Acid Sequence	Theoretical Mass/Observed Mass (Da)
STP1	10.393	GPDGR	500.51/500.43
STP2	12.096	GADIVA	544.60/544.55
STP3	14.827	GAPGPEMV	756.87/756.84
STP4	17.168	AGPM	374.46/374.39
STP5	17.583	GAEGFIF	739.82/739.76

**Table 4:** Radical scavenging activity of five antioxidant peptides (STP1–STP5) from gelatin hydrolysate of skipjack tuna (*K. pelamis*) bone.

No.	Half Elimination Ratio (EC_50_, mg/mL)			
	DPPH Radical	Hydroxyl Radical	Superoxide Anion Radical	ABTS Cation Radical
STP1	2.49 ± 0.12 ^a^	1.21 ± 0.08 ^a^	1.48 ± 0.12 ^a^	1.07 ± 0.07 ^a^
STP2	0.57 ± 0.03 ^b^	0.25 ± 0.02 ^b^	0.52 ± 0.03 ^b^	0.41 ± 0.03 ^b^
STP3	1.93 ± 0.11 ^c^	0.64 ± 0.05 ^c^	0.68 ± 0.05 ^c^	0.85 ± 0.06 ^c^
STP4	1.66 ± 0.09 ^d^	0.49 ± 0.03 ^d^	1.22 ± 0.08 ^d^	1.68 ± 0.11 ^d^
STP5	0.30 ± 0.04 ^e^	0.32 ± 0.03 ^b^	0.48 ± 0.03 ^b^	0.21 ± 0.03 ^e^

All data are presented as the mean ± SD of triplicate results. ^a–e^ Values with the same letters indicate no significant difference of different samples at the same radicals (*p* > 0.05).
